# Relationship of Exercise Capacity and Left Ventricular Dimensions in Patients with a Normal Ejection Fraction. An Exploratory Study

**DOI:** 10.1371/journal.pone.0119432

**Published:** 2015-03-10

**Authors:** Markus Meyer, Rachel K. McEntee, Iwan Nyotowidjojo, Guoxiang Chu, Martin M. LeWinter

**Affiliations:** Division of Cardiology, University of Vermont College of Medicine, Burlington, Vermont, United States of America; University of Central Florida, UNITED STATES

## Abstract

**Objectives:**

Extreme endurance exercise is known to be associated with an enlargement of the left ventricular (LV) chamber, whereas inactivity results in inverse changes. It is unknown if these dimensional relationships exist in patients.

**Methods:**

We analyzed the relationship of exercise capacity and LV dimension in a cohort of sequential patients with a normal ejection fraction undergoing stress echocardiography. In a total of 137 studies the following questions were addressed: (a) is there a difference in LV dimensions of patients with an excellent exercise capacity versus patients with a poor exercise capacity, (b) how is LV dimension and exercise capacity affected by LV wall thickness and (c) how do LV dimensions of patients who are unable to walk on a treadmill compare to the above groups.

**Results:**

Patients with a poor exercise capacity or who are unable to physically exercise have a 34 percent smaller LV cavity size when compared to patients with an excellent exercise capacity (p<0.001). This reduction in LV chamber size is associated with concentric LV hypertrophy and a reciprocal increase in resting heart rate. In addition, cardiac output reserve is further blunted by chronotropic incompetence and a tachycardia-induced LV volume reduction. In conclusion the relationship of exercise capacity and cardiac dimensions described in extreme athletes also applies to patients. Our exploratory analysis suggests that patients who cannot sufficiently exercise have small LV cavities.

## INTRODUCTION

In response to external demands the myocardium undergoes adaptive changes. This process has been termed cardiac plasticity and changes at the cellular and macroscopic levels have been documented under various environmental conditions [1]. The most common and best understood cardiac adaptation is concentric left ventricular remodeling, which most commonly develops in response to pressure overload due to hypertension and aortic stenosis [2,3].

The adaptive changes that occur with endurance exercise and deconditioning are not as well understood but have been described in the exercise physiology literature [4–6]. Adaptive eccentric remodeling with left ventricular chamber enlargement is well documented in endurance athletes such as long-distance cyclists [4]. Extreme physical inactivity induced by bed rest or zero-gravity conditions in healthy subjects has been demonstrated to rapidly reduce left ventricular chamber volumes and mass [5,6]. It is unknown if analogous exercise phenotypes are found in a general clinical population with less extreme physical activity and inactivity.

Changes in left ventricular mass and volume have been documented in select clinical populations that can be assumed to have a reduced exercise capacity, namely morbidly obese patients [7] and patients with severe pulmonary diseases [8–10]. In morbidly obese patients with and without heart failure a tendency towards eccentric left ventricular remodeling in the group of patients with heart failure symptoms was explained by an increase in the cardiac workload [7]. Several studies describe small left ventricular chamber dimensions in patients with COPD [8–10] and patients with end-stage pulmonary arterial hypertension are reported to develop left ventricular atrophy, that is probably the result of a left ventricular workload reduction [11,12]. All adaptive changes in the left ventricular dimensions volume have to be considered in the context of physiological aging, which is generally accompanied by smaller LV chamber volumes [13].

Although population or disease-based studies provide interesting structural insights into the remodeling of the left ventricle they typically do not directly relate left ventricular dimensions to exercise capacity, which is the primary objective of this study. Based on the exercise physiology findings and a large patient study that confirmed that poor exercise capacity is associated with diastolic dysfunction [14] it could be speculated that exercise capacity should also be reflected in the left ventricular dimensions. We hypothesized that poor exercise capacity in patients with a normal ejection fraction should be associated with a smaller left ventricle and chamber volumes, irrespective of the underlying cause or clinical presentation. Because this initial analysis suggested that an increase in left ventricular wall thickness significantly contributed to our findings we also explored the impact of left ventricular wall thickness on left ventricular volumes and exercise capacity.

## METHODS

The primary objective of this exploratory study was to determine if there are principal associations between exercise capacity and left ventricular dimensions in the population of adult patients referred for clinical stress testing. A secondary objective was to explore the effects of wall thickness on LV volumes and exercise capacity. To study these relationships we retrospectively analyzed a sequential cohort of patients undergoing Bruce-protocol based stress-echocardiography [15,16]. We also evaluated the left ventricular structure of patients who are unable to perform treadmill exercise and therefore underwent pharmacological echocardiographic stress testing.

### Patient Population

We used our imaging database of digitally stored stress echocardiograms obtained between 2009 and 2011. All studies were initiated by the treating physician and clinically indicated. Patients were referred for a variety of indications including chest pain, dyspnea on exertion/shortness of breath, coronary artery disease, and other (percentages of each shown in Tables 1–3). Exclusion criteria were EF <50%, baseline or stress induced wall motional abnormalities, clinically significant (≥moderate) valvular disease and poor echocardiographic image quality. The database was screened in reverse chronological order according to the date of study. The following patient characteristics were tabulated: test indication, cardiovascular risk factors, reason for test termination, gender, age, pre-test blood pressure and heart rate, body weight and height, medications, exercise duration, estimated metabolic equivalents of tasks (METs) and NYHA functional class. The University of Vermont Institutional Review Board has reviewed and approved this study. The data analysis was anonymous and no consent was required. A total of 137 studies were included in the analysis.

### Group Comparisons

In order to gain insight into the relationship between exercise capacity and LV chamber dimensions we selected groups of patients based on the following additional criteria:

**Poor exercise capacity versus excellent exercise capacity**. We first compared LV dimensions of patients with poor exercise capacity, defined as the ability to exercise less than 5 minutes on a standard Bruce treadmill protocol with patients with excellent exercise capacity, defined as the ability to exercise more than 15 minutes on the protocol. For both groups 20 studies were included in the analysis.

**Normal LV wall thickness versus increased LV wall thickness**. Since our initial analysis revealed a highly significant difference in wall-thickness at the extremes of exercise capacity we explored the effects of wall-thickness on LV volumes and exercise capacity. This analysis was performed in patients that were reported to have a normal exercise capacity on the clinical stress test interpretation. Inclusion criteria with either a *normal* septal wall thickness, (≤ 9mm in women and ≤ 10mm in men, n = 28), or *more than a mild increase* in wall thickness, (> 12mm regardless of gender, n = 21) [17]. All patients in this group reached more than 85% of the maximum predicted heart rate.

**Inability to exercise**. At our institution, dobutamine stress echocardiography is only performed in patients who are unable to walk on a treadmill. We hypothesized that this group of patients might represent a further physiologic and/or anatomic extreme of the poor exercise group. 48 patients were analyzed in this group.

### Echocardiography

**Fig 1:**
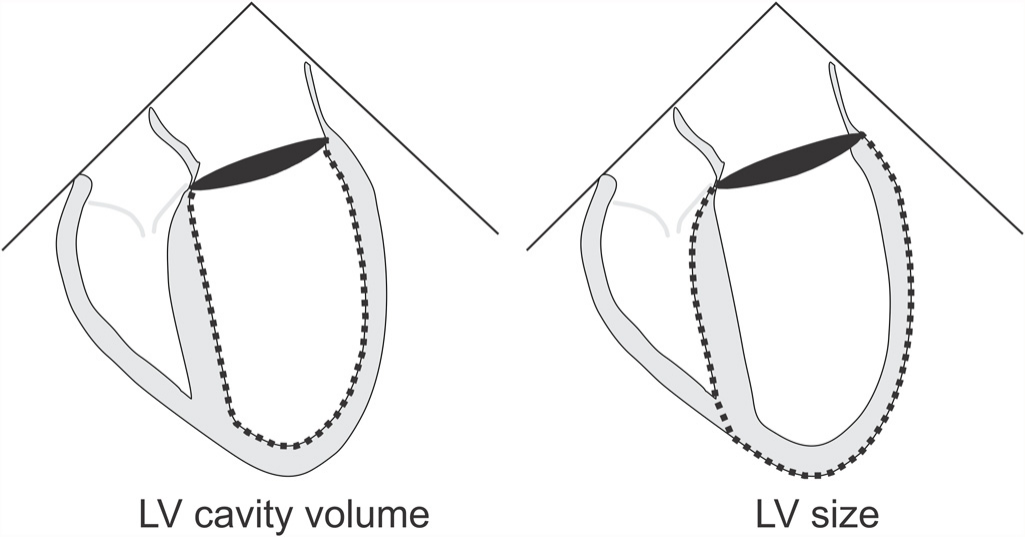
LV size and LV cavity volumetric assessment from the apical 4-chamber view.

All included studies had a sufficient endocardial definition (with or without echocardiographic contrast) to allow for an optimal assessment of LV dimensions and volumes. All studies were evaluated by two experienced readers. LV mass was calculated using the linear method (Devereux equation) and LV chamber volumes were calculated using the biplane method of disks (modified Simpson’s analysis) according to guideline recommendations [17]. LV chamber volumes were used to calculate ejection fraction and cardiac output by multiplying the volume-derived stroke volume by the echocardiography-documented heart rate. Total LV size using the biplane method of disks was determined by epicardial tracing to include the myocardium, as shown in Fig. 1. Where indicated, LV dimensions and volumes were normalized using height-based allometric indexing to minimize gender-differences and eliminate body weight-dependent underestimations of LV mass index in overweight subjects as discussed in the guidelines [17]. Optimal 2-D transthoracic echocardiographic parasternal long and short axis views in addition to apical two-chamber and four-chamber views were recorded [17,18]. To ascertain the reliability of our wall-thickness selection criteria we also determined the average LV wall thickness (AWT) in the parasternal short axis view. AWT was calculated by subtracting the endocardial radius from the epicardial radius derived from the short axis LV area calculation (A = r^2^π).

### Stress Test

Patients on the treadmill underwent a standardized Bruce treadmill protocol [15]. Images were obtained both at rest and at peak exercise following guideline recommendations [16]. In patients who underwent pharmacologic echocardiographic stress testing, intravenous dobutamine was delivered by an infusion pump starting at 5 mcg/kg/min for 3 minutes. The infusion rate was stepwise increased to 10, 20 and then 40 mcg/kg every 3 minutes until at least 85% of the maximum predicted heart rate was reached [16]. Images were captured at baseline, intermediate rate steps and at peak heart rate.

### Data Analysis and Inter-observer Variability

A power analysis using preliminary data of patients who exercised less than 5 minutes or more than 15 minutes revealed 80% power to detect a difference with group sizes of 20 using 2-sample t-tests with a two-tailed 5% Type I error level. Accordingly, we aimed to enroll at least 20 patients per group. LV end-diastolic and end-systolic volumes from the dobutamine stress test (rest and peak stress) and treadmill stress test (rest and peak stress) were plotted against the respective heart rates. The stress-induced change in LV volume was calculated in each patient and expressed as the change in LV volume (in ml) over a change in heart rate of 50 beats per minute. Between and within group comparisons were performed using Student’s t-tests. A two-sided Fisher’s exact test was employed for categorical data. P-values <.05 were considered to be statistically significant. Values are reported as mean ± standard deviation. An analysis of inter-observer variability of LV volumes resulted in a good correlation, with r = 0.88 and a typical error (standard deviation of the differences divided by the square root of 2) of 5 mL (95% CI, 6 to 18 mL). Statistical analysis was performed using IBM SPSS 18.0.0 software.

## RESULTS

### Exercise Capacity and Left Ventricular Dimensions

Patients in the poor exercise capacity group were able to exercise an average of 3 minutes and 39 seconds, whereas patients in the excellent exercise capacity group exercised for 16 minutes and 25 seconds. The estimated metabolic equivalent of tasks (METs) was 5±1METs in the poor exercise capacity group and 16±1METs in the excellent exercise capacity group. This translates to NYHA functional classes 3–4 and 1, respectively.

There were multiple significant differences in the baseline characteristics between groups as shown in Table 1. The poor exercise capacity group was older, had a higher body mass index and a significantly higher blood pressure. In addition, resting heart rate was significantly higher in the group with poor exercise capacity (difference of 14 beats per minute, p<.001). There was also a significant difference in the test indications; 60 percent of the patients with poor exercise capacity had dyspnea as the test indication, whereas this was only the case in 5 percent in patients with excellent exercise capacity (p<.001). Finally patients with poor exercise capacity had higher rates of hypertension and diabetes mellitus and were more likely to take ACE/ARB’s, calcium channel blockers and diuretics (p <.05 for all). Notably, there was no difference in the use of beta receptor antagonists between the groups.

**Table 1:** Excellent versus Poor Exercise Capacity.

	Excellent (NYHA 1)	Poor (NYHA 3–4)	
n = 20	n = 20	p <
Clinical Data	Age (years)	46±16	70±11	0.001
	Female (%)	4 (20)	10 (50)	n.s.
	BMI (kg/m^2^)	25±4	32±7	0.001
	BP (mmHg)	115/83	141/77	0.001/ n.s.
	HR (min^-1^)	59-±10	73±11	0.001
Test Indication	Dyspnea (%)	1 (5)	12 (60)	0.001
	Chest Pain (%)	10 (50)	4 (20)	n.s.
	CAD (%)	5 (25)	1 (5)	n.s.
	Other (%)	4 (20)	3 (15)	n.s.
Cardiovascular Risk Factors	Hyperlipidemia (%)	6 (30)	12 (60)	n.s.
	Current smoker (%)	2 (10)	2 (10)	n.s.
	Diabetes mellitus (%)	1 (5)	9 (45)	0.01
	Hypertension (%)	5 (25)	17 (85)	0.001
Medications	Betablocker (%)	5 (25)	7 (35)	n.s.
	ACEI / ARB (%)	4 (20)	12 (60)	0.05
	CCB (%)	0 (0)	7 (35)	0.01
	ASA (%)	9 (45)	3 (15)	n.s.
	Statin (%)	7 (35)	9 (45)	n.s.
	Diuretic (%)	1 (5)	8 (40)	0.05
Baseline Echocardiography	Septal Wall (mm)	8.1±0.1	12.7±0.3	0.001
	Posterior Wall (mm)	7.2-±0.1	10.6±0.2	0.001
	LVEDD (mm)	56±8	49±9	0.05
	LV mass (gr)	156±43	215±59	0.005
	LV mass index (gr/m^2.7^)	34±8	57±17	0.001
	EDV (mL)	97±20	55±18	0.001
	ESV (mL)	36±11	18±10	0.001
	EF (%)	63±8	68±13	n.s.
	LV Size (mL)	215±43	152±41	0.001

**Fig 2:**
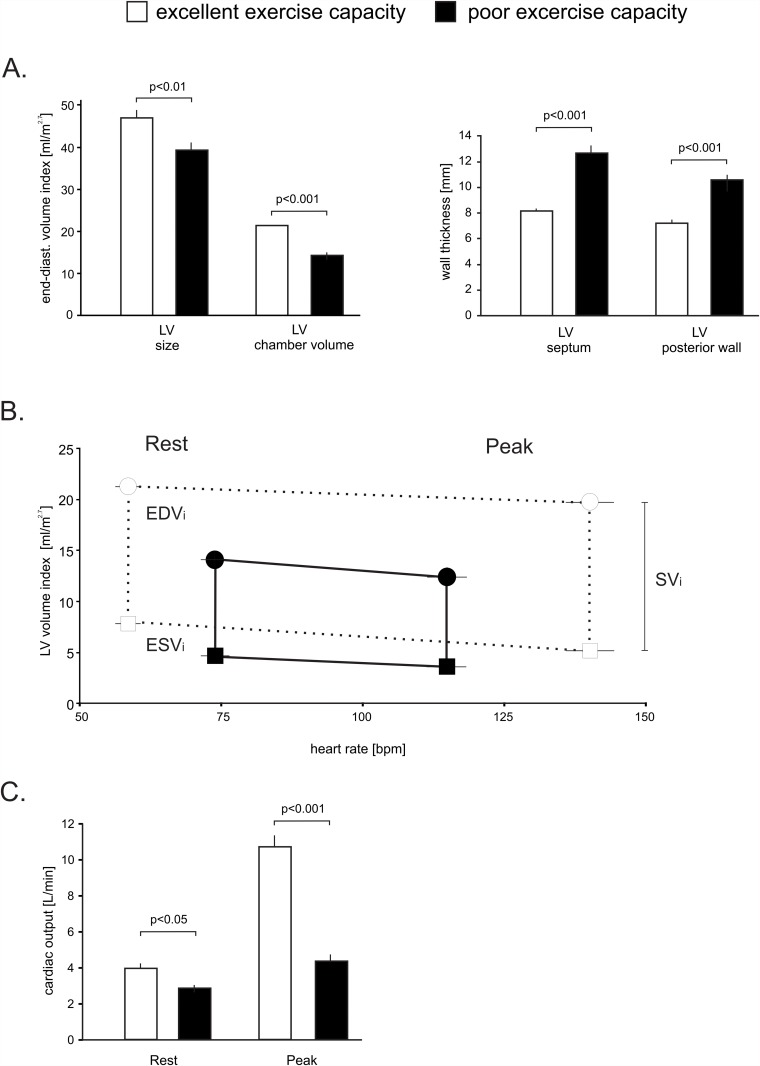
LV Volume and heart rate relationship in patients with a normal EF in patients with poor and excellent exercise capacity. Panel A demonstrates the differences in resting total LV size and LV chamber volume. The septal and posterior wall thickness was significantly increased in patients with poor exercise capacity. Panel B demonstrates the relationship of heart rate and indexed LV chamber volume at rest and with peak exercise. Circles: end-diastolic volumes, squares: peak systolic volumes. The vertical lines equal the stroke volume index. Panel C depicts cardiac outputs at rest and peak exercise in both groups. Error bars ±SE.

There were significant differences in cardiac dimensions as shown in Table 1 and Fig. 2. In the group with a poor exercise capacity, the non-indexed and indexed LV end-diastolic chamber volume was 44 and 34 percent smaller (both p<.001), and the septal and posterior LV wall was increased by 57 percent and 48 percent respectively (both p<.001), to suggest concentric remodeling. Accordingly, LV mass was significantly higher in patients with a poor exercise capacity, whereas the external LV dimensions were smaller. Total LV size, which includes the myocardium as shown in Fig. 1, that was 30 percent (non-indexed) and 16 percent (indexed) smaller (p<.001, p<.01).

In addition to the anatomic differences, physiologic differences were noted between the two groups. Despite a similar use of beta receptor antagonists patients with an excellent exercise capacity increased their heart rate by 99±24 bpm compared to 50±15 bpm in patients with very poor exercise capacity (p<. 001). The volumetric assessments also allowed us to estimate cardiac output. As shown in Fig. 2C, patients with an excellent exercise capacity nearly tripled their cardiac output with exercise whereas patients with a poor exercise capacity only increased their cardiac output by about 50% (p<.001). This marked difference was mainly driven by higher resting heart rates, reduced chronotropic response and additional reductions in end-diastolic volume at peak exercise.

### The Effect of LV Wall Thickness on Volumes and Exercise

We compared patients with a normal septal wall thickness to patients with a more than mild increase in septal wall thickness. All patients reached the target heart rate and were clinically judged as having a normal exercise capacity. The resulting groups were similar in age, body mass index, resting blood pressure and heart rate as shown in Table 2. Also, there were no significant difference in test indications and medications. However, patients with an increased wall thickness had higher rates of hypertension (p <.005).

**Table 2:** Normal versus Increased LV Wall Thickness.

		Normal Wall Thickness	Increased Wall Thickness	
		n = 28	n = 21	p<
Clinical Data	Age (years)	56±12	62±14	n.s.
	Female (%)	11 (39)	12 (57)	n.s.
	BMI (kg/m^2^)	28±4	30±6	n.s.
	Resting BP (mmHg)	127/76	131/77	n.s./n.s.
	Resting HR (min^-1^)	67±8	66±9	n.s.
Test Indication	Dyspnea (%)	6 (21)	5 (24)	n.s.
	Chest pain (%)	14 (50)	5 (24)	n.s.
	CAD (%)	5 (18)	7 (33)	n.s.
	Other (%)	3 (11)	4 (19)	n.s.
Cardiovascular Risk Factors	Hyperlipidemia (%)	22 (79)	14 (67)	n.s.
	Current smoker (%)	4 (14)	1 (5)	n.s.
	Diabetes Mellitus (%)	2 (7)	2 (10)	n.s.
	Hypertension (%)	11 (39)	18 (86)	0.005
Medications	Beta blocker (%)	4 (14)	7 (33)	n.s.
	ACEI/ARB (%)	9 (32)	11 (52)	n.s.
	CCB (%)	7 (25)	7 (33)	n.s.
	ASA (%)	10 (36)	12 (57)	n.s.
	Statin (%)	16 (57)	8 (38)	n.s.
	Diuretic (%)	2 (7)	6 (29)	n.s.
Baseline Echocardiography	Septal Wall (mm)	8.5±1.3	13.7±1.8	0.001
	Posterior Wall (mm)	8.0±1.2	11.9±1.8	0.001
	Mean Wall Thickness (mm)	7.5±1.3	12.7±1.8	0.001
	LVEDD (mm)	56±7	49±9	0.005
	LV mass (g)	174±49	251±87	0.001
	LV mass index (g/m^2.7^)	39±9	60±19	0.001
	EDV (mL)	87±29	70±23	0.05
	ESV (mL)	30±18	25±15	n.s.
	EF (%)	62±10	65±9	n.s.
	LV Size (mL)	188±54	206±62	n.s.

**Fig 3:**
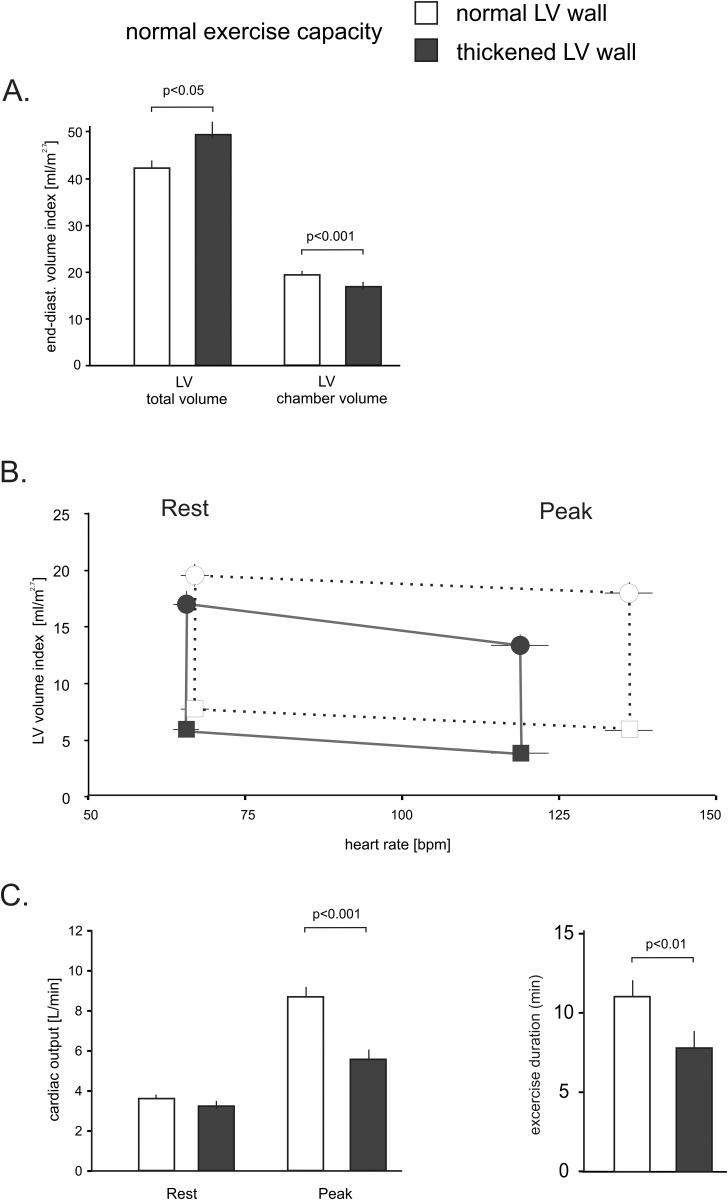
LV volume and heart rate relationship in patients with a completed stress test and normal or increased LV wall thickness. Panel A demonstrates the differences in the resting total LV size and LV chamber volume. Panel B demonstrates the relationship of heart rate and indexed LV volume at rest and with peak exercise. The exercise-dependent diastolic LV volume loss was significantly more pronounced in patients with an increased LV wall thickness. Circles: end-diastolic volumes, squares: peak systolic volumes. The vertical lines equal the stroke volume index. Panel C depicts cardiac output and exercise duration at rest and with exercise in both groups. Error bars ±SE.

In patients with an increased septal wall thickness the posterior and average LV wall thickness was increased by 48 percent and 69 percent respectively (both p<.001), whereas the LV end-diastolic diameter was reduced by 13 percent (all p<.005). Consistent with this finding, the indexed and non-indexed LV chamber volume was reduced by 13 percent and 19 percent (both p<.05). In patients with increased myocardial thickness the non-indexed and indexed LV mass were increased by 44 percent and 54 percent (both p<.001). The indexed total LV size, which includes the myocardium, was 17 percent larger in patients with an increased LV wall thickness as shown in Fig. 3A (p<.05).

Patients with a normal wall thickness increased their heart rate with exercise by 90±14 bpm, whereas subjects with increased wall thickness could only increase their heart rate by 67±14 bpm (p<.001). In patients with a normal wall thickness, the left ventricular end-diastolic volume did not significantly change with exercise, whereas a heart rate-dependent volume loss was observed in the group with a thickened myocardium, as shown in Fig. 3B. This change is manifest in a steeper slope of the line connecting end-diastolic volumes at rest and at peak exercise (-20±4mL/50bpm versus -7±2mL/50bpm, p<.05). Together, these variables significantly impaired cardiac output reserve in the group with an increased LV wall thickness. As a result, these patients increased their cardiac output by only 77 percent versus 250 percent in patients with a normal wall thickness (p<.01) as shown in Fig. 3C. There were also significant differences in exercise duration; patients with increased wall thickness exercised an average of 7 minutes and 53 seconds, whereas patients with a normal wall thickness exercised an average 11 minutes and 5 seconds (p<.01). Similarly, exercise capacity expressed in METs was 9±3 METs and 12±3 METs, respectively (p<.01). This confirmed that both groups were within the limits of a normal exercise capacity.

### Inability to Exercise on a Treadmill and Cardiac Dimensions

Pharmacological stress tests are performed in patients who cannot physically exercise. We hypothesized that irrespective of the reasons that prevented treadmill exercise this inability should be reflected in the LV phenotype. As shown in Table 3, these patients have similar baseline characteristics to the patients in the poor exercise capacity group. They are older, have an increased body mass index, hypertension, features of concentric LV remodeling and higher resting heart rates.

**Table 3:** Unable to Walk on Treadmill.

		n = 48
Clinical Data	Age (years)	64±14
	Female (%)	28 (58)
	BMI (kg/m^2^)	31±8
	BP (mmHg)	144/75
	HR (min^-1^)	75±11
Test Indication	Dyspnea (%)	11 (23)
	Chest Pain (%)	21 (44)
	CAD (%)	6 (12)
	Other (%)	17 (35)
Cardiovascular Risk Factors	Current smoker (%)	4 (8)
	Diabetes mellitus (%)	19 (40)
	Hypertension (%)	31 (65)
Medications	Betablockers (%)	16 (33)
	ACEI/ARB (%)	30 (63)
	CCB (%)	19 (40)
	ASA (%)	10 (21)
	Statin (%)	25 (58)
	Diuretic (%)	15 (31)
Baseline Echocardiography	Septal Wall (mm)	11.7±0.2
	Posterior Wall (mm)	11.0±0.2
	LVEDD (mm)	43±6
	LV mass (g)	166±59
	Mass Index (g/m^2.7^)	42±14
	EDV (mL)	59±34
	ESV (mL)	19±15
	EF (%)	70±11

**Fig 4:**
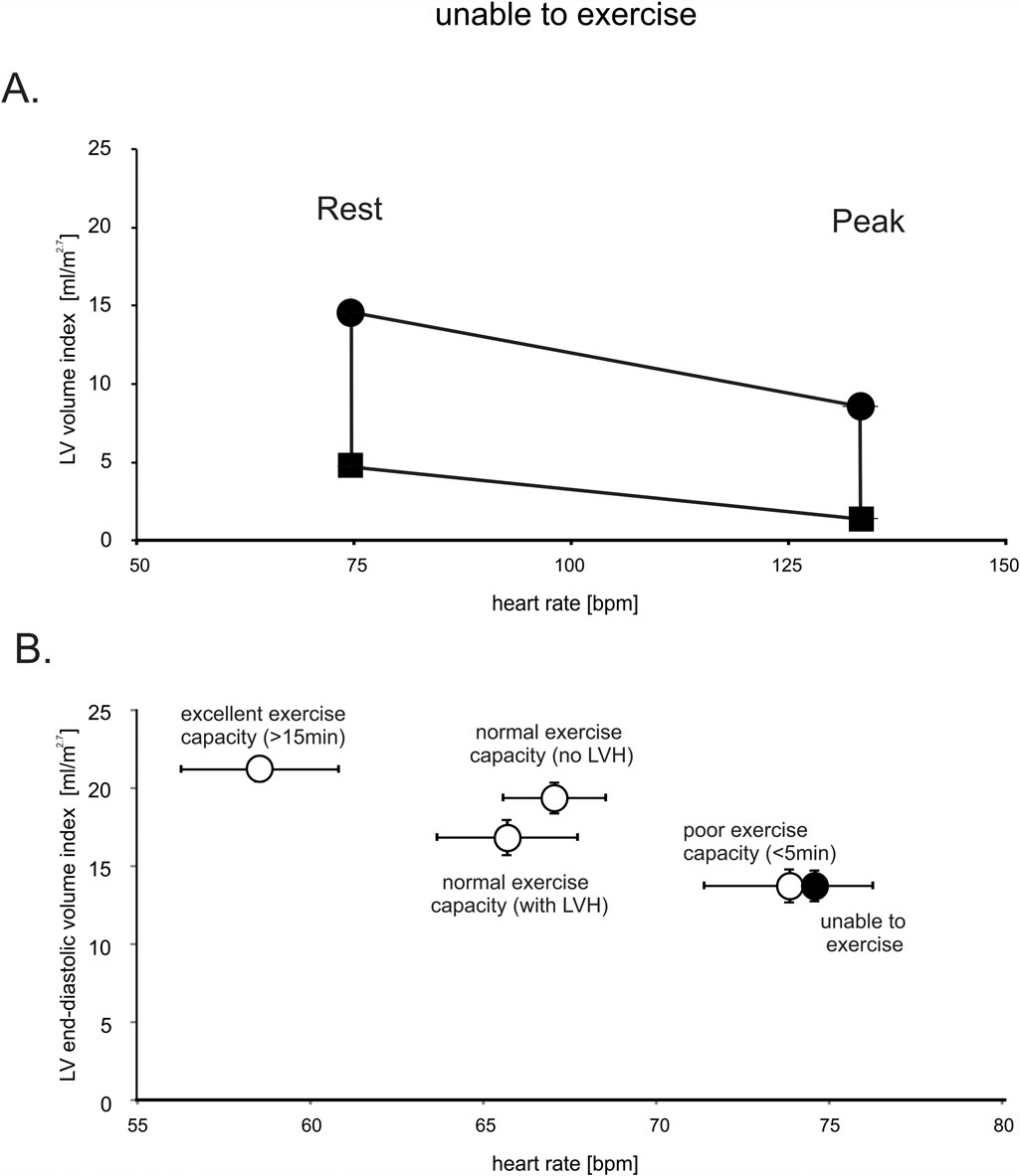
LV volume and heart rate relationship in patients with normal ejection fraction who are unable to walk on a treadmill. Panel A demonstrates the relationship of heart rate and LV volume index at rest and with peak exercise. Both systolic and diastolic chambers volumes decline substantially between rest and peak exercise which results in near LV cavity obliteration in systole. Circles: end-diastolic volumes, squares: peak systolic volumes. The vertical lines equal the stroke volume index. Panel B summarizes the relationship of heart rate and end-diastolic LV volume of all groups studied. Error bars ±SE.

Administration of dobutamine resulted in a pronounced diastolic and systolic volume loss, with near cavity obliteration at peak stress as shown in Fig. 4A. As a result of this pronounced reduction in the LV chamber size, cardiac output increased by only 34 percent. In patients who met left ventricular hypertrophy (LVH) partition values (>48g/m^2.7^ in men, >44g/m^2.7^ in women) we found a more pronounced end-diastolic volume loss. In patients with LVH, the slope of the volume loss was-28±5mL/50bpm compared to-13±5mL/50bpm in patients without LVH (p<.01). The same principal association was confirmed using our septal thickness criteria (p<.01).

Importantly, when directly compared to the other groups, left ventricular end-diastolic volume was identical to the group with a poor exercise capacity, as shown in Fig. 4B. Thus, in comparison to patients with a normal or excellent exercise capacity, patients who cannot sufficiently exercise also appear to have smaller LV cavity volumes.

## DISCUSSION

### Exercise Capacity and LV Dimensions

This cross-sectional exploratory analysis of a sequential cohort of patients undergoing stress echocardiography with a normal ejection fraction suggests a direct relationship between exercise capacity and left ventricular dimensions in a clinical population. Patients with a poor exercise capacity were found to have smaller left ventricular cavities and higher resting heart rates. In this observational study we can only speculate about cause-and-effect as we do not know if the observed reduction in chamber volume is in response to a sedentary lifestyle, aging or a preexisting phenotype associated with a low exercise capacity. However, rapid changes in left ventricular dimensions have been documented in healthy subjects exposed to bedrest or zero gravity conditions [5,6]. It appears therefore very likely that the observed differences in chamber volume are the result of an adaptive process of which hypertension-induced concentric remodeling appears to play an important role.

Our principal finding of a reduced LV cavity size was confirmed in the analysis of a diverse group of patients who were deemed unable to walk on a treadmill and were therefore referred for pharmacological stress testing. This particular result suggests that it may not matter *why* patients are unable to exercise; inactivity, for whatever reason, is associated with a small LV chamber size. Although small LV cavity sizes are typically encountered in patient with hypertension-induced concentric remodeling, as seen in this study, LV volume reductions are also reported in situations where the LV workload is severely reduced [12]. In our second analysis we explored the specific impact of wall thickness on LV volumes in patients who were considered to have a normal exercise capacity. This data suggests that patients with a seemingly normal exercise capacity but an increase in LV wall thickness are better able to maintain their LV chamber volume by an increase in the external dimensions of the LV. The finding that an increased LV wall thickness is still associated with a reduced cardiac output reserve and a lower exercise capacity mirrors changes seen in athletes, where individuals who performed non-endurance strength exercise such as weightlifting tend to have thicker left ventricular walls [19,20]. Pluim et al. compiled the literature on athletes in a meta-analysis examining this concept and conclude that although there seems to exist an “endurance trained heart” and a “strength trained heart,” adapted to handle high volume loads versus pressure loads respectively, this concept is not absolute but exists on a continuum [21].

The assessment of LV chamber volumes using the biplane method of discs is a recommended component of a transthoracic echocardiogram [17]. Because the measurement of the LV volume integrates both LV chamber size and the presence of concentric remodeling, it may be able to predict exercise capacity. After appropriate partition values have been further defined, an LV-dimension based “fitness-estimate” may present an additional opportunity to discuss the beneficial effects of physical exercise with patients as it is well established that physical fitness portends prognostic information. A recent longitudinal study examining the relationship between fitness level in middle age and subsequent hospitalizations for heart failure or myocardial infarction concludes that low levels of fitness in early middle aged were associated with increased rates of hospitalization for heart failure later in life [22]. In support of this notion Fujimoto et al. reported that one year of walking based exercise training in initially sedentary seniors, culminating in about 30 minutes of exercise per day, increases cardiovascular fitness and cardiac output primarily through decreasing peripheral vascular resistance. However, they also document a significant decrease in resting heart rate and increase in stroke volume index [23]. Importantly, the same investigators demonstrated that older individuals who exercise throughout their lives maintain normal LV dimensions [24], which supports our assertion, that differences in cardiac phenotype observed in this analysis are predominately maladaptive. In addition, structured exercise is so far the only intervention that appears to provide a clinical benefit in patients with heart failure and preserved ejection fraction, which tends to be associated with smaller LV cavity volumes and concentric remodeling [25,26].

### Variables Limiting Cardiac Reserve

As cardiac output is the product of heart rate and stroke volume, it is essential to discuss the impact of these variables on our findings.

**Heart rate**. Despite the fact that heart rate is by far the most important contributor towards cardiac output reserve in healthy individuals [27], relatively little attention has been given to the effects of heart rate on ventricular size, pump function and exercise capacity in patients. Our data demonstrate that resting heart rates are elevated in patients with a poor exercise capacity. This alone will inherently reduce cardiac output reserve. In addition these patients have chronotropic incompetence, or an inability to increase the peak heart rate sufficiently with exercise. This is a well-established observation in patients with heart failure with preserved ejection fraction (HFpEF) and an attempt has been made at correcting this deficit with rate-adaptive pacemaker therapy [28]. However, this trial was stopped prematurely and no information is available if the already enrolled patients derived a clinical benefit.

**Chamber volume**. Stroke volumes are generated by changes in chamber volume throughout the cardiac cycle and a smaller cavity size is typically associated with a smaller stroke volume [5,6]. This also implies that patients with smaller left ventricular cavities will more quickly develop a increased contractility-induced cavity obliteration. In resting patients, the stroke volume reducing effect of a smaller ventricular cavity appears to be compensated by an increase in resting heart rate but functions to reduce cardiac reserve during exercise as discussed above. Considering the dimensional restraints, only a significant exercise-induced increase in diastolic chamber volume (which conceivably could be accomplished by a shape change from an ellipsoidal to a spherical LV geometry) could improve stroke volumes since the normal pericardium precludes significant LV expansion [29]. This effect may underlay the exercise-induced 13 percent increase in LV cavity size in healthy subjects who underwent nuclear imaging during bicycle exercise [27].

The impact of walking and treadmill exercise, on LV size and volume are surprisingly not known. Our analysis suggests that the cavity size, at best, is maintained with treadmill exercise in patients with excellent exercise capacity, whereas patients with increased wall thickness experienced a substantial *loss* in end-diastolic volumes with exercise. Clinical studies that have evaluated the LV volume effect of various forms of exercise can be categorized as studies using upright and recumbent bicycle exercise or pacemaker-mediated tachycardia. In patients with hypertensive heart disease or in patients with diastolic dysfunction these studies have yielded conflicting results. In a recumbent bicycle ergometer thallium study, Cuocola et al. report that hypertensive patients whose ejection fraction did not increase adequately with peak exercise had a significantly greater LV mass [30]. In line with other bicycle ergometer studies, end-diastolic LV volumes were either unchanged or slightly increased with exercise [31,32]. When pacemakers are used to induce tachycardia, LV chamber volumes typically decrease. In a study that compared patients with concentric LVH to normal subjects Liu et al observed similar reductions in stroke volume at a pacing rate of 150/min [33]. At a pacing rate of 120/min Westermann and Wachter found that LV end-diastolic volumes and stroke volumes decreased substantially in patients with HFpEF but found them to be unchanged in control subjects [34,35]. Because prolonged myocardial relaxation has been documented in patients with HFpEF, it is indeed conceivable that rising heart rates could lead to a progressive impingement on relaxation [36,37]. This would manifest as a heart rate-dependent reduction in end-diastolic and stroke volumes that would blunt cardiac output reserve or, in the extreme, even reduce cardiac output. However, other factors such as inadequate arterial vasodilation with exercise and impaired left ventricular filling in the elderly and patients with diastolic dysfunction will also influence the LV volume in less predictable ways [38]. Tachycardia-induced reductions in left ventricular filling pressures could reduce the filling of the LV with *pacing-induced or pharmacological tachycardia* [35]. However, this later mechanism is clearly not playing a role with physical exercise where LV filling pressures are known to disproportionally increase in patients with diastolic dysfunction [39].

### Limitations

Similar to athletic and sudden-deconditioning studies, this exploratory analysis can only be used to provide initial insights into the relationship between left ventricular dimension and fitness at the extremes of the patient population that requires confirmation from larger prospective studies. Ideally such a study should include an assessment of diastolic function, which currently is not routinely assessed during stress echocardiography.

### Summary

In this exploratory analysis we found that the relationship between exercise capacity and LV chamber size observed in athletes and deconditioned subjects is also seen in patients. Patients with a poor exercise capacity or who are unable to exercise on a treadmill tend to have smaller and concentrically remodeled left ventricles with a reduced cavity volume. Thickening of the myocardium appears to result in an additional exercise-induced reduction in LV chamber volume. This volume reduction is potentiated by a higher resting heart rate and chronotropic insufficiency which both limit cardiac reserve. This is information is depicted in our heart rate-volume diagrams that provide a straight-forward visualization of all variables relevant to cardiac output.
